# A Method for Quantifying Mechanical Properties of Tissue following Viral Infection

**DOI:** 10.1371/journal.pone.0042197

**Published:** 2012-08-03

**Authors:** Vy Lam, Tarin Bigley, Scott S. Terhune, Tetsuro Wakatsuki

**Affiliations:** 1 Biotechnology and Bioengineering Center, Medical College of Wisconsin, Milwaukee, Wisconsin, United States of America; 2 Department of Microbiology and Molecular Genetics, Medical College of Wisconsin, Milwaukee, Wisconsin, United States of America; 3 Department of Physiology, Medical College of Wisconsin, Milwaukee, Wisconsin, United States of America; 4 InvivoSciences LLC, Madison, Wisconsin, United States of America; German Primate Center, Germany

## Abstract

Viral infection and replication involves the reorganization of the actin network within the host cell. Actin plays a central role in the mechanical properties of cells. We have demonstrated a method to quantify changes in mechanical properties of fabricated model three-dimensional (3D) connective tissue following viral infection. Using this method, we have characterized the impact of infection by the human herpesvirus, cytomegalovirus (HCMV). HCMV is a member of the herpesvirus family and infects a variety of cell types including fibroblasts. In the body, fibroblasts are necessary for maintaining connective tissue and function by creating mechanical force. Using this 3D connective tissue model, we observed that infection disrupted the cell’s ability to generate force and reduced the cumulative contractile force of the tissue. The addition of HCMV viral particles in the absence of both viral gene expression and DNA replication was sufficient to disrupt tissue function. We observed that alterations of the mechanical properties are, in part, due to a disruption of the underlying complex actin microfilament network established by the embedded fibroblasts. Finally, we were able to prevent HCMV-mediated disruption of tissue function by the addition of human immune globulin against HCMV. This study demonstrates a method to quantify the impact of viral infection on mechanical properties which are not evident using conventional cell culture systems.

## Introduction

Fibroblasts are ubiquitously distributed throughout the body and are necessary for establishing, maintaining and altering connective tissue during development and in response to injury [1], [2]. Carrying out these functions depends upon the ability of fibroblasts to remodel the extracellular matrix (ECM) by exerting force transmitted through cell-cell and cell-ECM interactions [3], [4]. These mechanical properties are mediated by the cytoskeleton which includes the actin microfilament network [5], [6]. Actin filaments transmit traction and contractile force generated by a myosin molecular motor and links to the ECM and focal adhesions. This force can be altered through diverse mechanisms including directly disrupting actin polymerization [7] or upon altering cellular signal transduction pathways [8].

Dysregulation of mechanical properties of connective tissue is associated with disease states including fibrosis in a variety of organs [1], [9]. Three-dimensional (3D) engineered tissues have been used to study fibroblast physiology by reconstituting a natural environment *in vitro* [10]–[13]. Recently, methods have been introduced to measure mechanics of model connective tissue [13]. This includes separately quantifying active cell-mediated contraction by fibroblasts and passive contribution mediated by the ECM. In this report, we apply the approach to quantifying the impact of viral infection on mechanical properties of tissue.

Infection and replication requires viral-mediated reorganization of the cellular cytoskeleton which includes the actin network [14]. Rearrangement of actin filaments occurs during infection by herpesviruses. Human cytomegalovirus (HCMV) is a member of the beta-herpesvirus family and infection causes disease throughout the world [15]. Acute HCMV infection is life threatening in immunologically immature or immunocompromised individuals including transplant recipients, AIDS patients, and neonates [15], [16]. Like all herpesviruses, primary infection is accompanied by a life-long persistent or latent infection with periods of reactivation. In addition to acute disease, persistent HCMV infection has been associated with cardiovascular disease including atherosclerosis, restenosis and transplant vascular sclerosis [17], [18].

HCMV can infect and replicate in a broad range of human cell types including macrophage, fibroblast, endothelial, smooth muscle and epithelial cells [19]. Infected fibroblasts and fibroblast-derived myofibroblasts have been implicated to participate in the efficient spread between bone marrow stromal and myeloid progenitor cells [19], [20]. During lytic replication [15], HCMV virions fuse with cellular membranes delivering the viral nucleocapsid, tegument proteins [21] and numerous RNAs [22], [23] into the host cell. The tegument proteins and viral RNAs establish a permissive environment for replication [24]. Delivery of the HCMV nucleocapsid to the nucleus depends upon intact microtubule [25] and intermediate filament networks [26]. The depolymerization of actin microfilaments has been observed during the early stages of infection [27]–[29]. It remains to be determined how HCMV infection influences the physiological activities of fibroblasts as an essential component in connective tissue function.

In this study, we have demonstrated a method to measure the impact of infection on the mechanics of 3D model connective tissue using HCMV.

## Results

### HCMV Infection of Model 3D Connective Tissue

**Figure 1:**
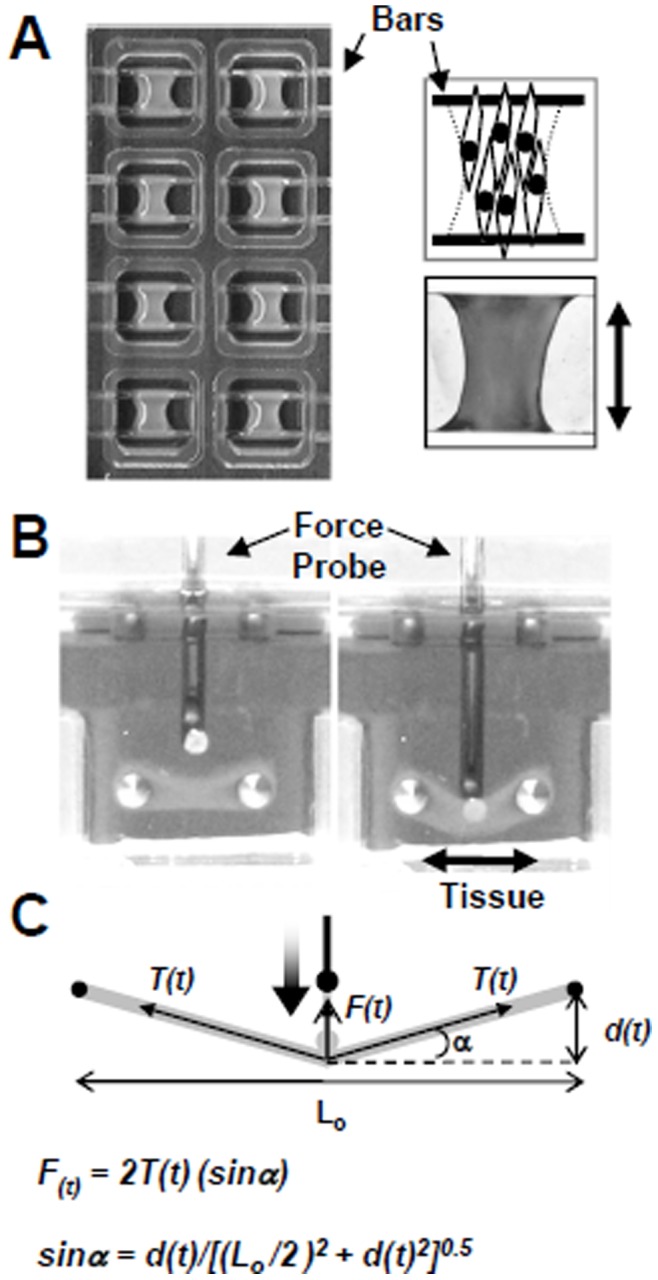
Model connective tissue system. (A) Tissue constructs were generated by mixing cells with neutralized collagen and adding to a tissue chamber (top view). The wells contain two horizontal stainless steel bars located 2 mm above the bottom of the well. The schematic and image of the formed tissue indicate cell and tissue orientation (double arrow). (B) A mechanical testing device measured tissue characteristics using a force probe (side view) that indents the tissues vertically and stretches it longitudinally. (C) The time-dependent resistance force measured by the probe, F(t), is expressed as a function of the probe’s movement, d(t), initial tissue length, L_o_, and the stress experienced by the tissue, T(t). The tissue is modeled as an isotropic linear elastic material and the active cell contractile force and matrix elasticity are obtained by least square fitting of F(t). The directions of F(t) and T(t) are represented by the solid arrows.

To investigate the impact of HCMV infection on fibroblasts and connective tissue mechanics, we used hydrogel tissue-constructs representing a model system for human connective tissue (Fig. 1A) [13], [30]. The tissues were generated by mixing primary human foreskin fibroblasts (HFF) with neutralized collagen. The solution was allowed to polymerize, trapping the cells within the collagen matrix. The entrapped cells at ∼2.7×10^5^ cells/well continue to divide, remodel the extracellular matrix (ECM) over 3 days and compact the tissue by cell traction force. As shown in Figure 1A, the fibroblasts and ECM attach to two stainless steel bars and cells align within the tissue with the orientation indicated by an arrow.

To measure the mechanical properties of the tissues, a robotic system containing a force probe (Fig. 1B) is used to indent the tissues. Values representing active cellular contraction by fibroblasts and passive contribution of the ECM can be determined by analyzing force data from the vertical stretching of the tissue constructs as described by Marquez *et al.* [13]. Briefly, the total resistance force experienced by the downward moving probe is modeled as a trigonometric function of the tissue force T(t), the probe movement distance d(t) (Fig. 1C), and the initial tissue length L_o_ (Fig. 1C). The tissue is then modeled as an isotropic linear elastic material where the total force on the probe, F(t), is equal to the sum of the active cellular force, and this increases linearly with tissue strain while the passive elasticity of the tissue increases exponentially. Although the model tissue allows quantification of force, the relevance of this model to what actually occurs *in vivo* has yet to be established.

**Figure 2:**
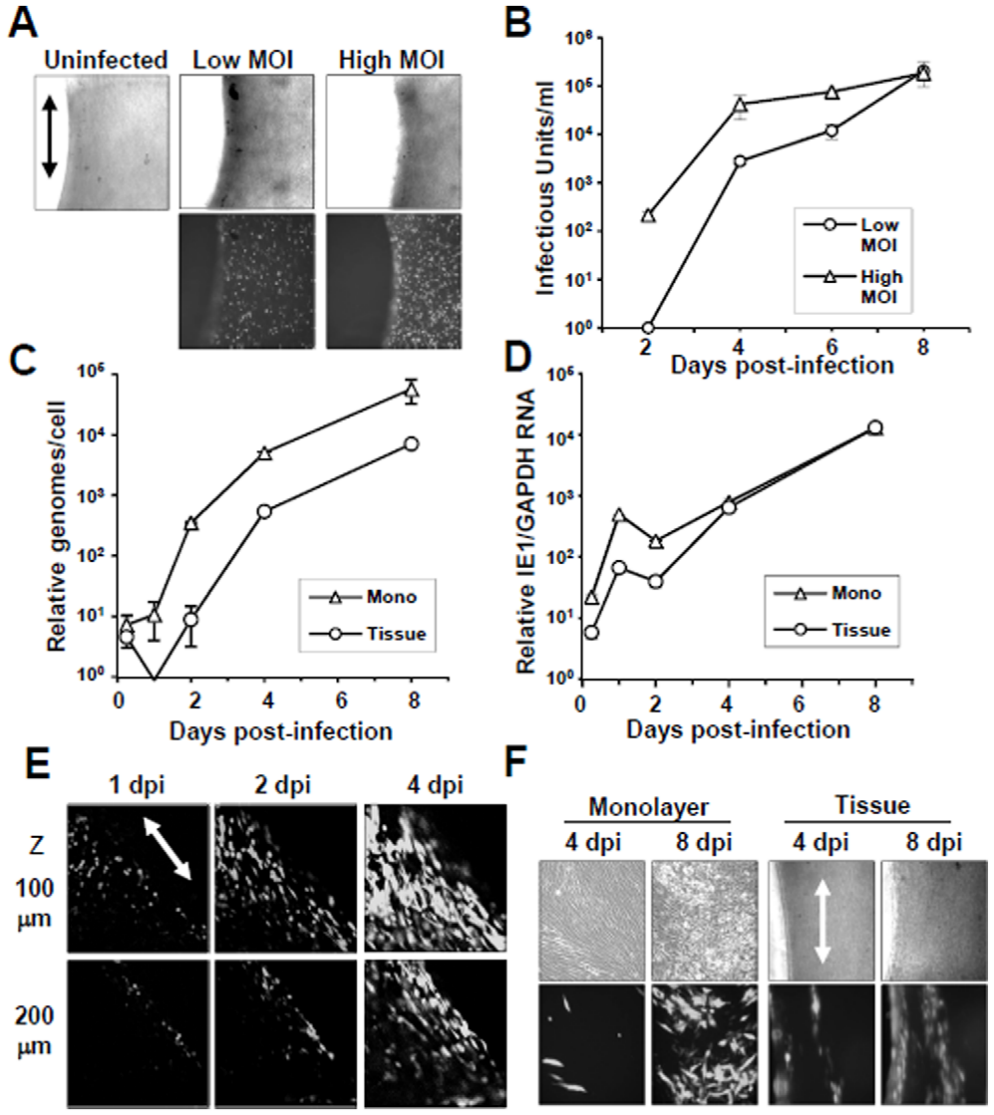
HCMV infects primary fibroblasts constrained within tissue. (A) Tissues were mock infected or infected using HCMV strain AD169 expressing the green fluorescent protein (ADgfp) at a multiplicity of either 1 or 10 pfu per cell. Infection was monitored by GFP fluorescence at 96 hpi. The orientation of the elongated fibroblasts and tissue is indicated by the arrow. (B) Growth kinetics of ADgfp measured using culture media from tissue wells. Titers were determined by infectious units per ml of media. (C) Approximately 1×10^6^ fibroblasts grown in monolayer cultures or in tissues were infected using ADgfp at an MOI of 0.1. Total DNA was harvested and HCMV genomes were quantified by qPCR using HCMV-specific primers. Quantities were normalized to the levels of cellular DNA using primers to beta-actin. (D) HCMV IE1 RNA expression was assayed by qRT-PCR using sequence-specific primers and RNA harvested from each culture. Quantities were normalized to the levels of GAPDH RNA within each sample. (E) Tissues were infected using ADgfp at an MOI of 10 and infected cells were detected using confocal microscopy. The location of the Z section was defined by the height from the bottom of the well with 100 µm being closer to the tissue surface. (F) Viral spread was monitored using ADgfp at an MOI of 0.1 and fluorescence in either monolayer cultures or tissues.

These tissues are three-dimensional relative to cells grown in two-dimensional monolayer cultures. To determine whether HCMV would be able to penetrate the ECM and infect the embedded fibroblasts, we incubated the wells with HCMV strain AD169 (ADgfp) encoding the green fluorescent protein (GFP) at a multiplicity of infection (MOI) of either 1 or 10 plaque forming unit (pfu) per cell. Because tissue mechanics are sensitive to serum levels, viral stocks were made by pelleting virus using centrifugation and resuspending the virus in serum-free media [31], [32]. Infections were completed by premixed virus with conditioned media obtained from the tissue wells. At four days post infection (dpi), images of the living tissues were captured using fluorescence microscopy (Fig. 2A). We observed GFP fluorescence in the embedded fibroblasts and increasing numbers of GFP-positive cells were seen at the higher MOI (Fig. 2A).

To determine whether infection of embedded fibroblasts could produce and release progeny virions, we completed a viral growth curve using media collected from wells infected at an MOI of 1 or 10. The titers were determined by quantifying HCMV IE1 antigen-positive cells per ml on monolayer cultures of HFFs. Increasing amounts of infectious virus were detected in the culture media from the tissues over time and in proportion to the input MOI (Fig. 2B).

### Delayed Onset of HCMV Replication in 3D Tissues

To determine if HCMV replicates with similar kinetics within fibroblasts growing in model 3D tissues as compared to monolayer cultures, we infected ∼1×10^6^ cells in either environment at a low MOI using ADgfp. We compared both HCMV DNA replication and IE1 RNA expression. Total DNA was isolated from cells in either monolayer or tissue culture and viral genomes were quantified using quantitative PCR. Although we observed similar levels of input genomes at 2 hpi (Fig. 2C), a delay in the onset of replication occurred in tissue samples. However, the overall rate of DNA replication was similar (Fig. 2C). To quantify RNA expression, total RNA was isolated from both environments and used for quantitative RT-PCR with primers specific to HCMV IE1 exon 4 sequence. We detected reduced levels IE1 RNA expression in the tissues occurring only at early times post-infection (Fig. 2D). Our data suggest that the virus has reduced accessibility to the fibroblasts embedded in the tissues yet replicates as efficiently as infection of cells cultured in monolayers.

To examine if there is reduced accessibility of virus to fibroblasts, we infected tissues at a high multiplicity and completed confocal microscopy on infected tissues. We evaluated viral penetration specifically at two separate Z sections in the same tissue location (Fig. 2E). We observed GFP fluorescence near the top surface as well as the edge of the tissues by 24 hpi (Fig. 2E). At 100 µm deeper into the tissues, we detected reduced numbers of GFP-positive cells which were restricted to the tissues edge (Fig. 2E). Tissues are approximately 800 µm thick and we observed increasing numbers of GFP-positive cells deeper into the tissue by 4 dpi (Fig. 2E). ADgfp preferentially spread horizontally in parallel to the orientation of the arranged fibroblasts in the tissues (Fig. 2F) as compared to that of the less organized fibroblasts in monolayer culture at 8 dpi (Fig. 2F). Our data demonstrate that HCMV has reduced access to cells in cells immobilized by ECM yet still replicates efficiently.

### HCMV Infection Decreased Mechanical Strength of Model Connective Tissue

**Figure 3:**
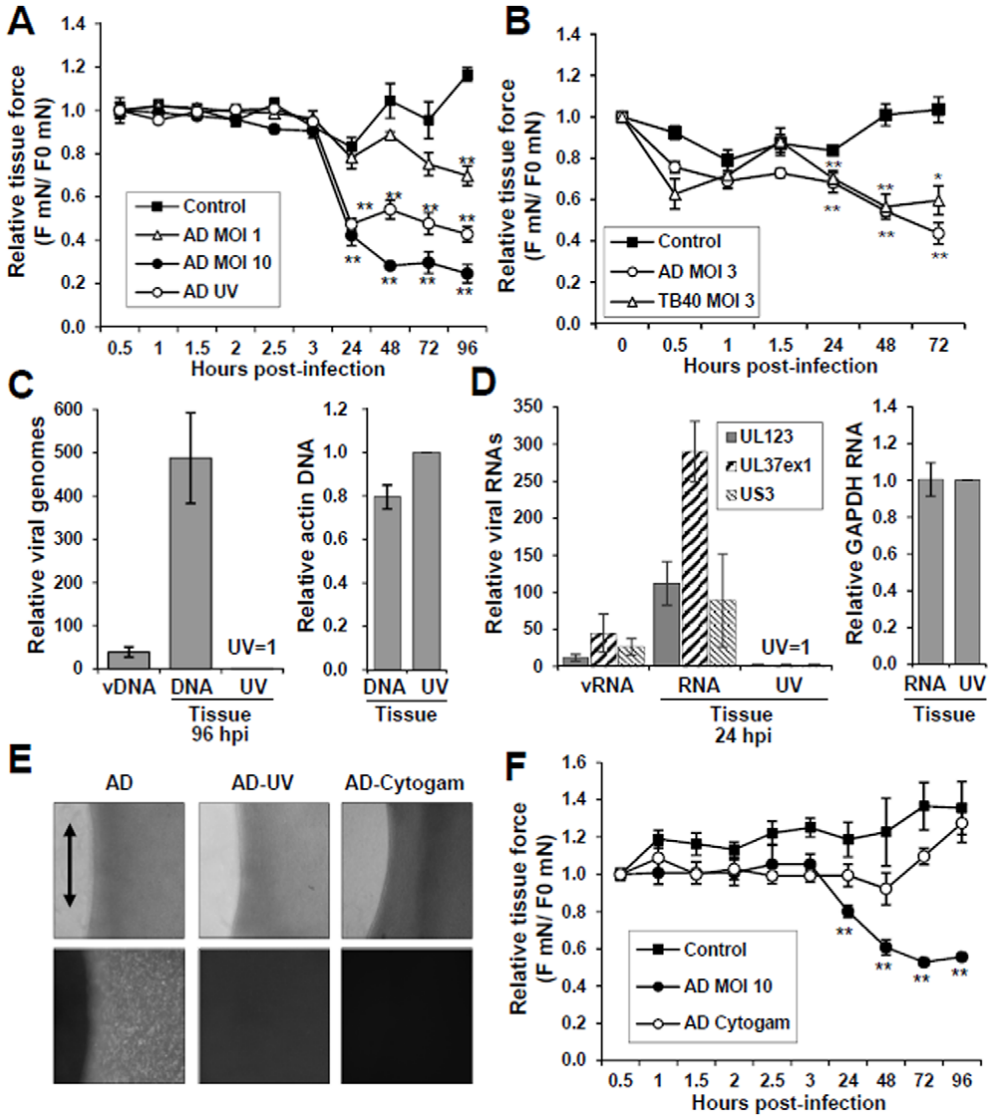
Disruption of the mechanical properties of tissues upon exposure to HCMV. (A) Tissues were m(C) Viral DNA was purified from infected tissues at 96 hpi as well as from the purified virus (vDNA) used in the inoculum and resuspended in equal volumes. DNA content was determined by qPCR using primers to HCMV or actin DNA. (D) Total RNA was isolated from tissue at 24 hpi as well as from the purified virus (vRNA) used in the inoculum and equal volumes were used for cDNA synthesis. Expression was determined using primers to immediate early genes in all samples and to cellular GAPDH in tissue samples. (E) Infection of the tissues was monitored by GFP fluorescence at 96 hpi for untreated ADgfp (AD), UV inactivated virus (AD-UV) and antibody neutralized virus (AD-Cytogam). Tissue orientation is indicated by an arrow. (F) Neutralization of ADgfp (MOI of 10) occurred by pre-incubating the inoculum in 5% Cytogam reagent (human immunoglobulin against CMV).ock infected or infected with ADgfp at MOI of 1, 10 or UV-inactivated virus (MOI of 10) using partially purified HCMV. Mechanical force measurements, F (mN), were taken beginning at 30 min postinfection (F_0_) and inoculum was removed after 3 hpi. The measurements are presented relative to F_0_. Values were calculated for the control at n = 6, infection at n = 4 and UV-treated virus at n = 2 (**p-value<0.01). (B) Tissues were infected with ADgfp or TB40/E at an MOI of 3 using purified virus (*0.01<p-value<0.05, **p-value<0.01).

The use of a 3D tissue model system allows for quantifying functional properties defined by the embedded fibroblasts and ECM. We determined the impact of HCMV infection on the mechanical properties of the tissues. Following fabrication, we infected the tissues with purified ADgfp at an MOI of 1.0 or 10.0. Mechanical force was measured beginning at 30 min post-infection and every 30 min up to 3 hr at which time the inoculums were removed and replaced with new media. We continued to measure tissue contractile force to 96 hpi while changing the media daily following each measurement. Prior to 3 hpi, we observed no detectable difference in force between uninfected control and HCMV infected tissues (Fig. 3A). However, a 49% drop in contractile force was observed at 24 hpi using an MOI of 10 compared to control (Fig. 3A). By 96 hpi, we observed a 79% decrease in force. At 96 hpi using a low MOI, we observed a 40% decrease in force (Fig. 3A).

HCMV strain AD169 has a narrow host range due to mutations within the UL128–131 gene locus and deletion of ∼15 kb region of the genome [33]–[37]. Clinical HCMV strain TB40/E does not have these disruptions and maintains a broad host range in cultured cells [38]. To determine the effect of TB40/E on tissue contraction, the tissues were infected with either ADgfp or TB40/E at an MOI of 3. Mechanical force was measured beginning at 30 min post-infection, and both ADgfp and TB40/E reduced tissue force by 43% and 59%, respectively at 72 hpi (Fig. 3B). Our data demonstrate that infection by HCMV resulted in an MOI-dependent mechanical disruption of the tissues.

### Disruption of Tissue Mechanics is Independent of Viral Immediate Early (IE) Gene Expression and DNA Replication

HCMV infection of fibroblasts results in cell lysis which is referred to as lytic replication. To determine if mechanical disruption can occur in the absence of lytic replication, purified virus was inactivated by UV treatment. At 24 hpi, addition of UV-inactivated virus resulted in a 43% drop in force relative to control (Fig. 3A). The drop in force increased over time and resulted in a 63% decrease by 96 hpi (Fig. 3A). These changes were similar to those observed using untreated HCMV.

To confirm that the UV-treatment inhibited the virus, we quantified viral DNA replication at 96 hpi in tissues. We observed a 500-fold decrease in viral genomes in tissues receiving UV-treated virus relative to untreated virus (Fig. 3C). HCMV genomes were quantified using equal volumes of input DNA between samples. Similar levels of actin DNA were observed between the two tissue samples, demonstrating equal amounts of input DNA in the quantitative PCR reaction (Fig. 3C). In addition, we detected fewer viral genomes in the UV-treated sample at 96 hpi relative to the input viral inoculum (Fig. 3C). To assess viral gene expression, we isolated total RNA from tissues receiving the untreated and UV-treated virus at 24 hpi along with total RNA from the input inoculum. We observed 100 to 300-fold lower levels of several IE RNAs in tissues receiving UV-treated virus (Fig. 3D) at similar levels of cellular GAPDH RNA within each sample (Fig. 3D). In addition, we detected lower levels of IE RNAs at 24 hpi in tissues receiving UV-treated virus when compared to the levels detected within the input inoculum (Fig. 3D). Finally, we did not see the appearance of GFP fluorescence in these tissues at 96 hpi (Fig. 3E). Our data suggest that the mechanical disruption observed in the tissues is due to HCMV viral particles and not the result of lytic replication.

Centrifugation of viral stocks results in the concentration of virus as well as cellular debris that accumulates during lytic replication. To confirm that the decrease in tissue force was the result of viral particles and not cellular debris, we pretreated the high MOI inoculum with the anti-HCMV human immune globulin reagent, Cytogam, to block infection. Cytogam is used as a prophylaxis to treat CMV disease. The addition of Cytogam to HCMV has been previously demonstrated to neutralize virus and prevent viral entry [39]. Following treatment with Cytogam, we did not observe GFP-positive fibroblasts at 4 dpi (Fig. 3E). More importantly, Cytogam prevented HCMV-mediated mechanical disruption (Fig. 3F). The uninfected control tissues were treated with the addition of 0.6% sucrose and 0.125% BSA, matching the stabilizing agents found in Cytogam (Fig. 3F) and, Cytogam alone did not influence uninfected tissue contraction (data not shown). These data demonstrate that the addition of HCMV viral particles is sufficient to induce tissue mechanical disruption.

### HCMV Particles Disrupted the Tissue Actin Microfilament Network Established by Fibroblasts

**Figure 4:**
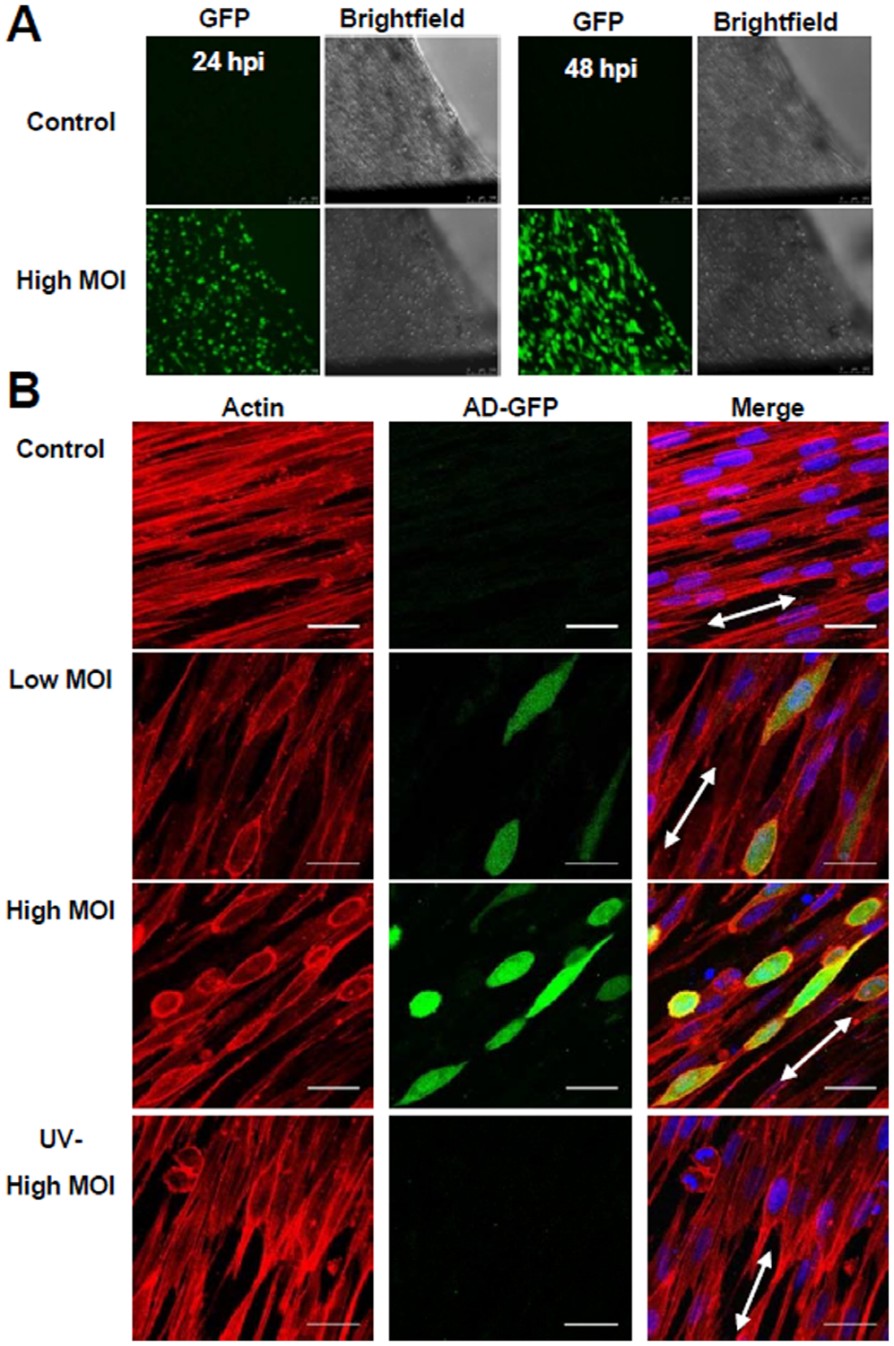
HCMV infection disrupts the actin microfilament network within the tissue. (A) Tissues were infected at an MOI of 10 and morphological changes of cells were observed by GFP fluorescence and brightfield microscopy of live cells at 24 and 48 hpi. (B) Model tissues were mock treated, infected using ADgfp or UV-treated ADgfp and fixed at 24 hpi. Changes in the actin microfilament network were assayed by fluorescence labeling. Actin was detected using phalloidin (red), infected cells were identified by GFP expression, and nuclei by DAPI (blue). Tissue orientation is indicated by the double arrow.

Actin microfilaments transmit mechanical force generated by actomyosin molecular-motors to the ECM and neighboring cells. Treatment of fibroblasts with Cytochalasin D, an inhibitor of actin polymerization, results in cells rounding up [40] and a significant drop in mechanical force [7]. Similar to Cytochalasin D treatment, we observed the majority of cells near the tissue surface rounding up following addition of ADgfp (Fig. 4A). The change coincided with the tissue mechanical disruption at 24 hpi (Fig. 3A).

In uninfected tissues, alignment of the fibroblasts can be observed by confocal fluorescence microscopy staining for the actin microfilament network (Fig. 4B). To determine if exposure of tissues to HCMV results in alterations to the microfilament network, tissues were infected using ADgfp, fixed and stained for filamentous actin at 24 and 48 hpi. We observed disruption of the actin filaments in cells of the infected tissue as compared to the untreated control (Fig. 4B). Addition of UV-treated virus also altered the actin network as well as cell morphology (Fig. 4B). However, the observed changes were less pronounced compared to untreated virus. Our results demonstrate that the addition of HCMV particles to model connective tissue disrupts the actin microfilament network established within the tissue.

## Discussion

In this report, we have demonstrated a method to quantify changes in the mechanical properties of model 3D connective tissue (Fig. 1A and 1B) following viral infection. Alterations in tissue physiology due to changes in intracellular signaling and cell-extracellular matrix (ECM) interactions can be quantified through measuring the mechanical properties of the tissues (Fig. 1C) [13]. In these studies, the addition of purified HCMV or UV-inactivated purified virus to the tissues resulted in a significant drop in the contractile force (Fig. 3A) [39]. Tissue mechanical disruption was observed for both the fibroblast-tropic HCMV strain, AD169, as well as the clinical strain, TB40/E (Fig. 3B) [38].

Past studies have demonstrated that reduction in tissue contractility occurs upon treating with the mycotoxin, cytochalasin D, which disrupts the cellular actin microfilament network in a dose-dependent manner [13], [32]. Microtubule disruption by nocodazole increases fibroblasts contractility [41] and intermediate filaments play limited roles in active fibroblast contraction (unpublished data). Upon the addition of purified HCMV, we observed disruption of actin microfilament network (Fig. 4B) and the drop in contractile force (Fig. 3A) at the same time, 24 hpi. Addition of UV-treated purified virus to the tissues also altered the actin network (Fig. 4B). However, the changes were less pronounced as compared to untreated virus (Fig. 4B). Alteration of the actin filament network has been observed during HCMV infection, and it remains to be determined if this is a requirement for infection or a consequence [28], [29], [42].

The 3D tissues used within these studies more closely resembles an *in vivo* microenvironment for cells in connective tissues (Fig. 1) [13]. Fibroblasts are embedded in the tissues and constrained by ECM. The tissue constructs are approximately 0.8 mm in thickness (Fig. 1B) [13] with a single cell layer at roughly 0.015 mm (Fig. 4B). Addition of HCMV to the tissues (Fig. 2A) resulted in a productive infection as observed by the release of infectious virus into the medium (Fig. 2B). To compare viral replication between cells growing in monolayers and 3D tissues, we quantified changes in both viral DNA replication (Fig. 2C) and RNA expression (Fig. 2D). We observed delayed but not inhibited HCMV IE1 gene expression and genome replication within the tissues as compare to monolayer cultures (Fig. 2C and 2D). It is conceivable that the 3D nature of the tissues is altering viral access and kinetics of entry into cells. Supporting this possibility, we observed reduced numbers of GFP-positive HCMV infected cells embedded within the tissues as compared to the tissue surface (Fig. 2E). This system will allow us to quantify the depth and rate of HCMV penetration into 3D tissues mimicking viral infection of human tissues.

Surprisingly, the addition of UV-inactivated virus to tissues was sufficient to cause mechanical disruption (Fig. 3A). The treated virus failed to replicate viral genomes (Fig. 3C), initiate IE gene expression (Fig. 3D) and express GFP that was encoded within the virus (Fig. 3E). In addition, we detected higher levels of viral genomes and RNAs within the input inoculum than that isolated from cells exposed to UV-treated virus (Fig. 3C and D). These observations suggest that the HCMV virions or component of virions are disrupting the actin network (Fig. 4B) and tissue force (Fig. 3A).

In general, binding of viruses to their target cells induces diverse actin-mediated responses. This includes movement of the bound viral particles to sites of entry, referred to as virion surfing, and induction of membrane ruffling and macropinocytosis [14]. For HCMV, binding of virions to their cellular entry receptors may be sufficient to alter cellular contractile force. HCMV has been demonstrated to interact with integrin, EGF and PDGF receptors during infection [29], [43]–[46]. These receptors are upstream of signal transduction pathways that regulate microfilament organization influencing tissue mechanics. In addition, two virally-encoded proteins have been previously demonstrated to alter filamentous actin [47], [48]. HCMV protein vMIA, coded by the immediate early UL37 gene, has been reported to disrupt actin stress fibers during infection [48], [49]. However, our observation that UV-treated virus disrupted tissue function (Fig. 3A) in the absence of gene expression (Fig. 4B) would suggest that vMIA is not involved. A second HCMV protein that influences the actin network is the G-protein-coupled receptor, US28 [47]. HCMV US28 is a component of virions [21], [50] and expression of US28 in the absence of infection alters actin stress-fiber formation [47], [51]. Regardless of the mechanism, we have demonstrated that the use of the antiviral immune globulin reagent, Cytogam, prevented CMV-mediated mechanical disruption (Fig. 3E and 3F).

We have demonstrated a method that will allow for the evaluation of events occurring during infection of 3D human tissues. The ability to quantify macroscopic physiological parameters such as tissue contractile force and matrix elasticity cannot be achieved using current *in vitro* 2D cell culture systems. This study demonstrated that HCMV disrupts the mechanical force established by human fibroblasts within tissues and it is conceivable that the resulting tissue damage may contribute to the diverse pathologies associated with persistent HCMV infection *in vivo*.

## Materials and Methods

### Cell Culture and Viruses

Human foreskin fibroblasts (HFF) were cultured in Dulbecco’s Modified Eagle’s Medium (DMEM) (Invitrogen, Carlsbad, CA) supplemented with 10% fetal bovine serum (FBS) (Invitrogen, Carlsbad, CA). Cells were used between passages 4 to 15. HCMV strain AD169 is a BAC-derived virus containing the green fluorescent protein gene (ADgfp) [52] and strain TB40/E [38], [53] is a BAC-derived virus containing the mCherry gene. Both viruses were propagated on fibroblasts. The mCherry version of the TB40/E BAC was kindly provided by Eain Murphy. Viruses were concentrated 10-fold by centrifugation through a sorbitol cushion (20% D-sorbitol, 50 mM Tris-HCl [pH 7.2], 1 mM MgCl_2_), pellets were resuspended in fresh serum-free DMEM, and titers were determined by using an immunofluorescence assay to quantify IE1-expressing cells [54]. Viral titers from growth curves were determined using the same method. UV-inactivated virus was obtained by treating the inoculum using 360 mJ/cm^2^ using a Spectrolinker UV crosslinker (Spectronics, Westbury, New York) as previously described [55]. Neutralized virus was prepared by incubating the inoculum with 5% neutralizing antibodies to HCMV (CytoGam, CLS Behring, King of Prussia, PA) prior to infection [39].

### Tissue Constructs

To make hydrogel tissue constructs, HFF cells were dissociated from culture plates by treating with 0.05% trypsin and the cells were then centrifuged at 1000 x g for 10 min. The trypsin solution was removed and the cell pellet was re-suspended in 10% FBS DMEM medium. This cell suspension was diluted in tissue solution to achieve a final concentration of 8×10^5^ cells per ml. The tissue solution consisted of 10% FBS DMEM containing 1 mg/ml of type 1 collagen (BD Biosciences, San Jose, CA). The solution was made by dissolving collagen in 0.02 N acetic acid, adjusting the pH to 7 by the addition of sodium hydroxide, and adding 5× DMEM to a final 1× DMEM concentration. To prevent premature collagen polymerization, the tissue solution was kept on ice until its distribution into MC-8 Mini-Construct Chambers (InvivoSciences, McFarland, WI) [13]. Each of these MC-8 chambers contains 8 separate tissue-forming wells with two built-in horizontal support bars. Tissue solution was aliquoted at 300 µl per well and then incubated for 30 min at 37°C and 5% CO_2_. After the incubation period, 350 µl of 10% FBS DMEM medium was added to each well and the molds were further incubated for 72 h. After this time, the chambers contained ∼1×10^6^ cells and the solution contracted to form tissues that span the support bars in the wells as observed in Figure 1A.

### Tissue Force Measurement

The high throughput mechanical testing device, Palpator (InvivoSciences, McFarland, WI), was used to quantify the contractility of the tissues [13]. The MC-8 molds were placed on the stage of the Palpator which automatically inserted a probe into each well and stretched the individual tissue. The probe was connected to a force transducer which measured the resistance force induced in the tissue in response to stretch and exported the values to a computer for recording. A custom Matlab algorithm [13] was used to process and analyze the force data to report a numerical parameter that is indicative of the active cell force in the tissue. While viral infections might reduce the active cell force anisotropically, the probe measured the tissue’s total force. Therefore, the algorithm treated it as an isotropic elastic material and computed an averaged force reduction. To obtain stable measurements of the tissue contractile force, it was necessary to precondition the tissues by stretching three times prior to actual force measurement [13]. Preconditioning was not necessary if subsequent force measurements were within 30 min of the previous stretch. Tissues resting in the incubator for 24 consecutive hours were always pre-conditioned before force measurement.

Prior to HCMV infection, tissues were first precondition stretched three times and then stretched a fourth time to measure background force. Following the four stretches, culture medium in replicate wells were removed and mixed with control medium, virus, UV virus, or Cytogam-treated virus and then redistributed back into the wells as described above. In case of infection medium and fibroblast-conditioned medium treatments, the medium in the wells were replaced with 500 µl of the perspective medium. Following infection, tissues were stretched every 30 min for 3 hr and then once each day for 4 days. Incubation medium in all the wells were changed to new 3% FBS DMEM at the end of the 3 hr of inoculation and every day following force measurement.

### Tissue Imaging

Infected and control tissues were fixed by incubating in 500 µl of 4% formaldehyde (Sigma, St Louis, MO) in PBS solution for 45 min. Tissues were then rinsed twice with 500 µl of PBS. Tissues were permeabilized by incubating in 500 µl of 0.1% Triton (Thermo Fisher Scientific Inc, Waltham, MA) solution in PBS for 15 min and then rinsed twice with 500 µl of PBS. To label the tissues, they were incubated in 1∶200 diluted Alexa 568 conjugated phalloidin (Invitrogen, Carlsbad, CA) in PBS with 800 nM of DAPI (Sigma, St. Louis, MO) for 45 min. Tissues were then rinsed twice with PBS. Labeled tissues were then cut from the support bars and sandwiched between a cover-glass slide and a cover glass for confocal microscopy. Images of the tissues were captured using a Leica SP5 confocal microscope (Leica Microsystems, Bannockburn, IL) with 63× water immersion objective. Alexa 568 was excited using the 543 laser and DAPI was excited using a MaiTai multi-photon laser. Images with lower magnification were captured using a HP Scanjet 3970 (Hewlett-Packard, Palo Alto, CA) or on a Nikon Eclipse TS100 microscope equipped with a mercury lamp and TRITC, FITC, and DAPI filter cubes (Nikon Instruments, Melville, NY).

### RNA and DNA Quantification

RNA levels from infected cells and tissues were quantified by real-time reverse transcription-PCR (qRT-PCR). Total RNA was harvested from either monolayer cultures or tissue constructs that had been infected using equivalent inoculums. In addition, RNA was harvested from the equivalent input inoculum as previously described [23]. RNA was isolated using Trizol reagent (Invitrogen, Carlsbad, CA), following the manufacturer’s instructions, resuspended in 30 µl of water and contaminating DNA was removed using DNA-free reagent (Applied Biosystems, Foster City, CA). Relative quantification was accomplished through two-step real-time RT-PCR, as previously described [54]. Briefly, cDNAs were synthesized using 1 µg of total RNA with SuperScript III reverse transcription reagents and random hexamers according to the manufacturer’s instructions in a 40 µl reaction volume (Invitrogen, Carlsbad, CA). Real-time PCR was completed using 1 µl of cDNA with SYBR green PCR master mix (Applied Biosystems, Foster City, CA) and primers specific to exon 4 of UL123 (5′-GCCTTCCCTAAGACCACCAAT-3′ and 5′-ATTTTCTGGGCATAAGCCATAATC-3′) (Integrated DNA technologies, Coralville, IA), or glyceraldehyde-3-phosphate dehydrogenase (GAPDH) (5′-CTGTTGCTGTAGCCAAATTCGT-3′ and 5′-ACCCACTCCTCCACCTTTGAC-3′). Quantities for unknown samples were defined relative to a standard curve consisting of 10-fold serial dilutions of a single sample and completed for each primer pair. For studies comparing RNA levels to input virion RNA, equal volumes from each sample were used in cDNA synthesis reactions. Primers were also used for US3 (5′-TTCCACTCGAAATAGGCTCCGC-3′ and 5′-CGAGAAACACTTTGTGAACGTGGG-3′) and UL37 exon 1(5′-ATCCTCTCCCGCCTTGGTTAAGAA-3′ and 5′-TCACCGTCAATTACGCCATGTTGG-3′). PCR quantification was performed using the 7900 HT Fast Real-Time PCR System (Applied Biosystems, Foster City, CA). DNA levels were also determined by real-time PCR. Infected monolayer cultures, tissues or viral inoculum samples were harvested and lysed in lysis buffer (400 mM NaCl, 10 mM Tris [pH 8.0], 10 mM EDTA, 0.1 mg/ml proteinase K, 0.2% sodium dodecyl sulfate [SDS]) at 37°C overnight. For all samples, DNA was extracted with phenol-chloroform, extracted again with chloroform, and precipitated with ethanol. Samples were resuspended in 50 µl water and 1 µl was quantified by real-time PCR analysis using primers specific for the UL123 gene or cellular ß-actin [54].
